# Development of Quinoxaline 1, 4-Dioxides Resistance in *Escherichia coli* and Molecular Change under Resistance Selection

**DOI:** 10.1371/journal.pone.0043322

**Published:** 2012-08-28

**Authors:** Wentao Guo, Haihong Hao, Menghong Dai, Yulian Wang, Lingli Huang, Dapeng Peng, Xu Wang, Hailan Wang, Min Yao, Yawei Sun, Zhenli Liu, Zonghui Yuan

**Affiliations:** National Reference Laboratory of Veterinary Drug Residues/MOA Key Laboratory of the Detection of Veterinary Drug Residues, Huazhong Agricultural University, Wuhan, Hubei, People’s Republic of China; Aligarh Muslim University, India

## Abstract

Quinoxaline 1, 4-dioxides (QdNOs) has been used in animals as antimicrobial agents and growth promoters for decades. However, the resistance to QdNOs in pathogenic bacteria raises worldwide concern but it is barely known. To explore the molecular mechanism involved in development of QdNOs resistance in *Escherichia coli,* 6 strains selected by QdNOs *in vitro* and 21 strains isolated from QdNOs-used swine farm were subjected to MIC determination and PCR amplification of *oqxA* gene. A conjugative transfer was carried out to evaluate the transfer risk of QdNOs resistant determinant. Furthermore, the transcriptional profile of a QdNOs-resistant *E. coli* (79O4-2) selected *in vitro* with its parent strain 79–161 was assayed with a prokaryotic suppression subtractive hybridization (SSH) PCR cDNA subtraction. The result showed that more than 95% (20/21) clinical isolates were *oqxA* positive, while all the 6 induced QdNOs-resistant strains carried no *oqxA* gene and exhibited low frequency of conjugation. 44 fragments were identified by SSH PCR subtraction in the QdNOs-resistant strain 79O4-2. 18 cDNAs were involved in biosynthesis of Fe-S cluster (*narH*), protein (*rpoA, trmD, truA, glyS, ileS, rplFCX, rpsH, fusA*), lipoate (*lipA*), lipid A (*lpxC*), trehalose (*otsA*), CTP(*pyrG*) and others molecular. The 11 cDNAs were related to metabolism or degradation of glycolysis (*gpmA* and *pgi*) and proteins (*clpX, clpA, pepN and fkpB*). The *atpADG* and *ubiB* genes were associated with ATP biosynthesis and electron transport chain. The pathway of the functional genes revealed that *E. coli* may adapt the stress generated by QdNOs or develop specific QdNOs-resistance by activation of antioxidative agents biosynthesis (lipoate and trehalose), protein biosynthesis, glycolysis and oxidative phosphorylation. This study initially reveals the possible molecular mechanism involved in the development of QdNOs-resistance in *E. coli*, providing with novel insights in prediction and assessment of the emergency and horizontal transfer of QdNOs-resistance in *E. coli*.

## Introduction

Quinoxaline-1,4-dioxides (QdNOs) have been introduced into animal production as potent antibacterial agents against most gram-positive and gram–negative pathogens since 1970s [1], [2], [3]. These drugs have been also widely used as growth promoters for decades [4], [5]. Carbadox, maquindox and olaquindox are the well known members of QdNOs [6]. Cyadox is a new member of QdNOs which exhibits better antibacterial activity, lower toxicity and higher efficiency on animal growth promotion [7], [8], [9], [10].

It is demonstrated that usage of olaquindox on pig farms may increase the emergence of olaquindox resistance in some pathogens [11], [12]. The bacteria with resistance to olaquindox are generally co-resistant to ampicillin, tetracycline or chloramphenicol [1], [13]. For the mechanism of QdNOs resistance, it is only known that plasmid-encoded multidrug efflux pump OqxAB confers resistance to ampicillin, chloramphenicol, ciprofloxacin and olaquindox in *E. coli* [14], [15]. This OqxAB located plasmid has a high frequency of transfer among enterobacterial pathogens (*Salmonella Typhimurium, Klebsiella pneumoniae, Kluyvera sp.* and *Enterobacter aerogenes*, *etc*) [14], [16]. It is rarely known how *E. coli* develop or acquire the QdNO’s resistance. For the concern of animal and human safety, it is required to be elucidated whether the selection pressure of QdNOs contributes the emergence of OqxAB and whether the resistant determinants would spread to other enterobacterial pathogens. During the development of QdNOs resistance, it is an urgent need to explore the novel molecular mechanisms involved in the emergency and transfer of QdNO’s resistance in pathogenic bacteria especially *E. coli*.

Since serial passage is a traditional way to predict the resistance development to some antibiotics [17], some QdNOs resistant *E. coli* strains selected by two-fold increasing concentration of olaquindox and cyadox *in vitro* and some clinical QdNOs-resistant *E. coli* strains isolated from swine farms in China where QdNOs have been used for a long time, were subjected to analyze the development of QdNOs resistance. Since conjugation is the most important way of horizontal transfer of some antibiotic resistance determinants in *E. coli* [18], the horizontal transfer of QdNOs-resistant determinant was then assessed by conjugative experiment. Since a prokaryotic SSH PCR cDNA subtraction approach developed by De Long is a technique that can be used to identify differentially expressed genes under given conditions [19], this approach was consequently carried out to investigate the novel mechanism beyond the OqxAB-mediated QdNOs resistance.

## Materials and Methods

### Drugs and Stock Solutions

Olaquindox (OLA), mequidox (MEQ), ampicillin (AMP), ceftiofur (CTF), tetracycline (TET), chlorotetracycline(CTE), chloramphenicol (CHL), florfenicol (FFC), trimethoprim (TMP), sulfamethoxazole (SMZ), ciprofloxacin hydrochloride (CIP) and colistin sulfate (CS) were supplied by China Institute of Veterinary Drug Control. Gentamicin (GEN), oxytetracycline (OTC), furazolidone (FZD) and rifampicin (RIF) were provided by National Institute for the Control of Pharmaceutical and Biological Products (Beijing, China). Carbadox (CAR) was purchased from Sigma Chemical Co. (St. Louis, MO) and cyadox (CYA) was kept in our lab. Stock solutions of the antimicrobials were prepared following the guidelines of the Clinical and Laboratory Standards Institute (CLSI, 2009) or the suppliers’ recommendations. Besides, stock solutions of CYA, CAR and FZD were prepared in DMSO; Stock solutions of OLA, MEQ and FFC were prepared in hot water; Stock solutions of CTF were prepared in phosphate buffer (pH 6.0, 0.1 mol/L). After vortexing, the solutions were sterilized by filtration through 0.22 µm pore size filters (Millipore) before storage in aliquots at −20°C. Solutions of ampicillin, tetracycline and oxytetracycline were prepared fresh on each occasion.

### Strains

The porcine *E. coli* 79–161 strain was purchased from China Institute of Veterinary Drug Control (Beijing, China). *E. coli* NK5449 (CGMCC NO. 1.1437) being resistant to rifampicin at 100 µg/ml and as conjugation recipient was purchased from China General Microbiological Culture Collection Center (CGMCC, Beijing). *E. coli* ATCC25922 used as quality control in MIC test was kindly donated by South China Agricultural University (Guangzhou, China). All the strains were cultured at 37°C in Luria-Bertani (LB) broth medium in aerobic incubator.

### In vitro Selection of High-level QdNOs Resistant Strains

The selection of high-level QdNOs-resistant strains was performed with ancestor strain *E. coli* 79–161 by serial transferring in LB broth containing two-fold increased concentrations of CYA or OLA. The selection concentrations of CYA and OLA ranged from 1/2MIC to 4MIC. Each step of transferring contained 5 passages of parent strain in broth with certain concentration of drug. Four increased concentrations of drug (1/2MIC, MIC, 2MIC and 4MIC) led to a total of 20 passages of parent strain. During each passage, an initial colony concentration of 10^4^∼10^5^ cfu/ml of parent strain was grown at 37°C for 24 h in aerobic incubator. Cultures obtained at 5^th^, 10^th^, 15^th^ and 20^th^ passages were stored at −20°C. To estimate the stability of resistance, the 20^th^ subcultures were serially transferred in drug-free LB broth for 10 times. For each passage, a drug-free culture (growth control) and an uninoculated medium sterility control were included.

### In vivo Isolation of Resistant *E.coli* from Swine

On swine farm in Wuhan Huaguoshan Livestock Company, six groups of swine were breeding with drugs (Group 1 with no drug, group 2 with 50 µg/ml olaquindox, group 3 with 50 µg/ml chlorotetracycline, group 4 with 50 µg/ml cyadox, group 5 with 150 µg/ml cyadox and group 6 with 250 µg/ml cyadox). The samples of anal swab were collected from each group of swine after 0, 45^th^ and 100^th^ day adding drugs to their basal diet. The *E. coli* strains were isolated and identified by the selective agar (MacConkey Agar) and classic biochemical test following the microbiology laboratory guidebooks published by ministry of health in China (2003). Species identification was confirmed using an ABI 3130 system (Applied Biosystem, USA). 1∼2 respective *E. coli* strains selected from each of the six groups were subjected to MIC determination as below. The experimental procedures involving animals in the study were approved by the Animal Care Center, Hubei Academy of Medical Sciences. All efforts were made to minimize suffering of animals.

### MIC Determination

MIC was determined by agar dilution antimicrobial susceptibility test according to the M7-A7 guideline of the Clinical and Laboratory Standards Institute (CLSI, 2006).Briefly, the density of *E.coli* suspensions was adjusted to equal the density of a 0.5 McFarland standard (1×10^8^ CFU/ml). After 10 fold dilution by MH broth, 1∼2µL of the diluted suspension was inoculated on prepared MH agar plates containing two-fold diluted concentration of each drug. The final concentration of strains was 10^4^CFU/point on drug-containing plates. The standard strain of *E. coli* ATCC25922 was used as quality control (QC) strain. Most of the QC range and susceptible breakpoint were cited from CLSI document M100-S19. The QC range of CIF was quoted from performance standard of cefotaxime and ceftriaxone. The QC range of FFC was quoted from performance standard of chloramphenicol. The resistance breakpoint of FZD (128 µg/ml) was quoted from performance standard of furantoin. The resistance breakpoints of CTF, FFC and CAR were cited from published paper [12]. Olaquindox resistance has been defined with a breakpoint of 64 µg/ml [1]. Acquisition of cyadox resistance was defined as an ≥4-fold increase of the MIC value comparing with its control parent strain [20].

### PCR Amplification of *oqxA* Genes

Colony PCR was proceeded to screen for *oqxA* genes in the selected *E.coli* strains. Based on the sequences of *oqxA* (GeneID 5962055) in *E. coli*, a pair of primers (OqxA-F 5′-GGGATAGTTTTAACGGTCGCATTG-3′ and OqxA-R 5′-TT CACGGGAGACGAGGTTGGT-3′) was designed to amplify 266-bp *oqxA* gene fragment. The amplification conditions comprised a denaturation step of 95°C for 5 min, followed by 32 cycles of 94°C for 30 s, 64°C for 30 s, 72°C for 1 min and a final extension for 10 min at 72°C. Amplicons were sequenced by Nanjing Genscript Biotechnology Limited Company (Nanjing, China).

### Conjugative Transfer of QdNOs-resistant Determinant

To investigate whether QdNOs-resistant determinant possess horizontal transfer risk, filter conjugation was carried out according to the method used by Yuan et al [21], in which induced high-level QdNOs-resistant strains were used as donor and *E. coli* NK5449 was used as recipient. The transconjugants was selected on LB agar plates containing 100 µg/ml olaquindox and rifampicin. The frequency of conjugation was calculated as following formula: CFU of transconjugant/CFU of donor. The donor, recipient and transconjugant were identified by comparison of their *Eco*RI RiboPrint® pattern with that in identification database [22]. Comparing to *E. coli* NK5449, the similarity of the RiboPrint® pattern was shown in percent.

### Preparation of mRNA

Before isolation of total RNA, driver and tester stains were grown in 50-ml drug-free Luria-Bertani (LB) broth till log phase. Briefly, overnight cultures were 100 fold diluted in 50-ml of LB broth, the culture were grown at 37°C with 250 rpm shacking. When the density of culture reached to 0.6∼0.8 OD_600_, the cells were harvested by centrifugation of 1-ml aliquots in a microcentriguge for 2 min. An RNeasy Protect Bacteria Mini Kit (Qiagen, Valencia, CA, USA) was used to lyse the cells and extract total RNA. The RNA was quantified by a light absorption at 260/280 nm. The purity and integrity of the RNA samples were verified by an electrophoresis in agarose gels using the 16S and 23S ribosomal bands as indicators. Samples of valid RNA were DNase treated with RQ1 RNase-free DNase (Promega, Madison, WI, USA). The DNA contamination was verified by PCR amplification of *rpoE* gene using the primers in Table 1. Reactions containing pure RNA preparations would fail to yield a product. The mRNA was isolated using a MICROBExpress bacterial mRNA enrichment kit (Ambion, Austin, TX, USA). Every 5 preparations for each substrate were pooled before ethanol precipitation to obtain at least 2 µg mRNA per preparation. The mRNA quantity was measured by absorbance at 260 nm, and quality was assessed on agarose gel.

**Table 1:** Primers used in SSH PCR cDNA subtraction and RT-qPCR.

Gene	Forward and reverse primer sequences	Melting temperature (°C)	Reference
16S rRNA (*rrsC*)	5′-GCTACAATGGCGCATACAAA-3′ 5′-TTCATGGAGTCGAGTTGCAG-3′	55.9	[69]
*rpoE*)	5′-TCTGGTTTCCCGCTATGT-3′ 5′-AGCTCCCGCAAGGTTATT-3′	51	This study
*rpsA*	5′-CTGGACGCAGTAGAAGACGG-3′ 5′-ACGGCACGACGAGAAACA-3′	60	This study
*mobA*	5′-TGTATCCGCCCGCCAAGA-3′ 5′-AAGGCTGTGACCTCAAACGAGA-3′	56.7	This study
*rpoA*	5′-GCAGTTAGCAGAGCGGACAG-3′ 5′-GGAAACCAACGGCACAAT-3′	62.0	This study
*rplF*	5′-AACACCGATAACCATTGAGT-3′ 5′-GCAGATAATACCCTGACCTT-3′	56.9	This study
*fusA*	5′-GCTGACGCAGAGCACCTT-3′ 5′-CGAATGGCACCAGAACCT-3′	59.1	This study
*fabI*	5′-CCAAGGTAGGAAAGGGTC-3′ 5′-AGTGCGATGTTGCCGAAG-3′	56.4	This study
*clpX*	5′-AAACGCCCTGACCAAGCA-3′ 5′-GGACGGCAGATCGTACATG-3′	54.8	This study
*atpD*	5′-CCAGGGTGTAACGATAGATG-3′ 5′-ACCACGAAATGACCGACT-3′	59.1	This study
*ubiB*	5′-GGGCAAGTATTTACAGCACA-3′ 5′-AACCAGGCGATCAGACCAC-3′	56.9	This study
*lolC*	5′-CTTCGGTCGTTTCGTCTCC-3′ 5′-GCTCAAAGCCGTTCATCACT-3′	63.5	This study
*secA*	5′-CTTCCCGTTTCTACCTGTC-3′ 5′-TTGGCAATCGCTTTAGTC-3′	54.0	This study
*fliC*	5′-GACGGTATTTCTGTTGCG-3′ 5′-TCTTCCAGGCGAGATTTG-3′	59.1	This study

### Prokaryotic SSH PCR cDNA Subtraction

cDNA synthesis, cDNA digestion, adaptor ligation, first and second hybridization, suppression PCR and clone screening were carried out following the protocols of De Long with slight modification on the PCR-select cDNA subtraction kit(Clontech) [19].

A RNA polymerase sigma factor *rpoE* (the primer in Table 1) was used to detect the quality of double-strand cDNA. The amplification conditions comprised a denatured step of 95°C for 5 min, followed by 32 cycles of 94°C for 30 s, 51°C for 30 s, and 72°C for 1 min and a final extension for 10 min at 72°C. Products were resolved by gel electrophoresis on a 2% (wt/vol) agarose gel. After second-strand cDNA synthesis, two cDNA synthesis reaction mixtures were pooled and purified with DNA fragment purification kit (Takara, Kyoto, Japan). Tester and driver cDNA were digested with *Rsa* I and purified also with DNA fragment purification kit (Takara).

To determine the efficiency of adapter ligation and subtraction, PCR amplification were performed according to the protocol in the PCR-Select cDNA subtraction kit by use of a housekeeping gene ribosomal protein S1 (*rpsA*) (the primer in Table 1). The amplification conditions comprised a denatured step of 95°C for 5 min, followed by cycles of 94°C for 30 s, 60°C for 30 s, and 72°C for 1 min and a final extension for 10 min at 72°C. For test of ligation efficiency, the cycles were 32. And for test of subtraction efficiency, the cycles were 18, 23, 28, 33 and 38.

After the PCR adaptor screen, 1, 2-clones were selected and submitted to Nanjing Genscript Biotechnology Limited Company (China) for sequencing directly. The sequences were aligned to available sequences in NCBI library by blastn algorithm (www.ncbi.nlm.nih.gov. BLAST). The homological genes were subjected into molecular annotation system (http://www.capitalbio.com/support/mas) or KEGG library for gene function annotation and pathway analysis.

### Reverse Transcription, Quantitative Real-time PCR (RT-qPCR)

4∼5 µg of DNase-treated total RNA (isolated as described above) was used to synthesize cDNA according the method of De Long [19]. Real-time PCR was carried out on Bio-Rad iQ™5 Multicolor Real-Time PCR Detection System, using SYBR® Premix Ex TaqTM (Takara) in a total volume of 25 µl: 12.5 µl SYBR® Premix Ex TaqTM, 1.0 µl template cDNA, 0.5 µl (final concentration 0.2 µM) or 0.25 µl (final concentration 0.1 µM) of each primer, and PCR-grade water to a final volume of 25 µl. Samples without template were set as negative control. At least 3 replicates were analyzed for each gene. Primer sequences were presented in Table 1.The fold change of selected genes was normalized to fold change of reference gene (16S rRNA gene). The relative expression was calculated according to the formula 2^−△△Ct^ [23], The data were considered for significant differences by one-way ANOVA using SPSS programme (P≤0.05).

## Results

### Resistance Phenotype of Selected Strains

Six representative strains (79O4-1, 79O4-2, 79O4-3, 79C4-1, 79C4-2 and 79C4-3) were obtained after stepwise selection by two-fold ascended concentration of olaquindox and cyadox. The variations of MICs during the selection procedure were presented in Table 2. Before selection, strains were susceptible to all the antibiotics tested. After 20 passages, the MICs of growth-control strains (79∼20) have no significant change. High-level olaquindox resistant *E. coli* strains (79O4-1, 79O4-2 and 79O4-3) with MIC ≥256 µg/ml were selected under the pressure of olaquindox. Cyadox resistant *E. coli* strains (79C4-1, 79C4-2 and 79C4-3) with MIC ≥128 µg/ml were obtained by stepwise selection of cyadox. Notably, *E. coli* strains selected by olaquindox exhibited reduced susceptibility to ampicillin, tetracycline, oxytetracycline, chloramphenicol, florfenicol, trimethoprim and ciprofloxacin (see Table 2). All of the three strains (79O4-1, 79O4-2 and 79O4-3) selected by olaquindox were cross-resistant to the four QdNOs tested, while the three strains (79C4-1, 79C4-2 and 79C4-3) selected by cyadox were only resistant to carbadox and cyadox. Compared to cyadox, olaquindox might provide more potential in selection of multidrug resistance and cross-resistance in *E.coli*.

**Table 2:** The MICs to 16 antibiotics of the strains selected by two-fold ascended concentrations of olaquinodox or cyadox *in vitro.*

strains	MIC to different antibiotics (µg/ml)															
	AMP	CTF	GEN	TET	OT	CHL	FFC	TMP	SMZ	FZD	CIP	CS	OLA	MEQ	CAR	CYA
selected by olaquinodox																
79O4-1	16*	1	0.25	64#	32#	8	8	8*	8	8	0.031*	1	256#	128#	64#	>128#
79O4-2	16*	2*	0.25	64#	32#	32#	32#	8*	8	8	0.031*	1	>256#	>128#	128#	>128#
79O4-3	8	≤0.5	0.5*	64#	32#	8	8	4*	8	16	0.031*	1	256#	128#	32#	>128#
selected by cyadox																
79C4-1	2	≤0.5	0.25	32#	32#	2	2	1	8	8	0.015	1	32	32	64#	>128#
79C4-2	2	≤0.5	0.25	32#	32#	4	4	1	8	8	0.015	1	16	16	8	>128#
79C4-3	4	≤0.5	0.5*	32#	32#	8	8	1	8	4	0.015	1	32	32	64#	>128#
Growth control strain																
79∼20	4	≤0.5	0.125	2	2	4	4	1	8	8	0.008	1	16	16	16	32
QC standards																
ATCC25922	2	0.125	0.5	2	2	2	4	2	8	8	0.008	1	8	4	4	16
QC range	2–8	0.03–0.12^f^	0.25–1	0.5–2	0.5–2	2–8	2–8	0.5–2	8–32	4–16^h^	0.004–0.015	0.25–1	NA	NA	NA	NA
Breakpoint	32	8	16	16	16	32	16	16	512	128	4	NA	64	4MIC	32	4MIC^k^

Note:. * MIC ascends 4-fold or more, but not reaching breakpoint;

# MIC reach breakpoint. NA Not available.

Before and after adding olaquindox, chlorotetracycline and cyadox into swine diet, 21 representative *E.coli* strains were isolated from 6 groups of swine by anal swab. From the data shown in Table 3, there is no significant change of the MIC before and after usage of QdNOs. Before usage of drug, the 7 *E.coli* strains selected at 0 day exhibited resistance to olaquindox with MIC ≥64 µg/ml. Notably, the 3 *E.coli* strains from the control group is naturally resistant to olaquindox with MIC = 64 µg/ml or 256 µg/ml.

**Table 3:** Olaquindox MICs and *oqxA* containing in the clinical strains isolated from pigs before and after usage of growth promoters.

Time for drugaddition (days)	group	Usage of Drugs and concentrationin swine diet	strains	Olaquindox MIC(µg/ml)	*OqxA (*+*positive*, - *negative)*
0 day	1	control	E0-1	64	+
	2	50 µg/ml olaquindox	E0-2	64	+
	3	50 µg/ml chlorotetracycline	E0-3	128	+
	4	50 µg/ml cyadox	E0-4	64	+
	5	150 µg/ml cyadox	E0-5	128	+
	6	250 µg/ml cyadox	E0-6-1	128	+
	6	250 µg/ml cyadox	E0-6-2	128	+
After 45 days	1	control	E45-1	256	+
	2	50 µg/ml olaquindox	E45-2	256	+
	3	50 µg/ml chlorotetracycline	E45-3	128	+
	4	50 µg/ml cyadox	E45-4	256	+
	5	150 µg/ml cyadox	E45-5	128	+
	6	250 µg/ml cyadox	E45-6-1	64	+
	6	250 µg/ml cyadox	E45-6-2	64	+
After 100 days	1	control	E100-1	256	+
	2	50 µg/ml olaquindox	E100-2	256	+
	3	50 µg/ml chlorotetracycline	E100-3	128	+
	4	50 µg/ml cyadox	E100-4	32	+
	5	150 µg/ml cyadox	E100-5	64	-
	6	250 µg/ml cyadox	E100-6-1	128	+
	6	250 µg/ml cyadox	E100-6-2	256	+

### *oqxA* Gene Mediated Resistance

All of the *in vitro* selected stains presented in Table 2 were negative for *oqxA* gene products, indicating that other mechanisms except for OqxAB mediated the resistance in the selected strains. However, 95% (20/21) *E.coli* strains isolated from swine farms were positive for *oqxA* gene (see Table 3).The *oqxA* positive strains are isolated before and after the usage of QdNOs drugs on pig farm. No relationship can be observed between the occurrence of *oqxA* gene and usage of QdNOs drugs. The *oqxA* gene positive strains always exhibited resistance to olaquindox except for E100-4 strain which carried *oqxA* gene but is susceptible to olaquindox (MIC = 32 µg/ml).

### Frequency of Conjugal Transfer

Frequencies of conjugal transfer for each strain were exemplified in Table 4. The conjugation frequencies of the three *E. coli* donors were at 10^−10^ CFU/donor. Before conjugation, the recipient is susceptible to the four QdNOs but resistant to rifampicin, the donors are resistant to the four QdNOs but susceptible to rifampicin. After conjugation, the transconjugants were resistant to both four QdNOs and rifampicin (see Table 4). The recipient and transconjugants share similar RiboPrint® pattern with the similarity >90% (see Table 4), confirming that QdNOs-resistant determinant had been horizontally transferred by conjugation in *E. coli* strains.

**Table 4:** Transfer frequency and specific assessment of conjugation**.**

Backgroud	strains	Transfer frequency (transconjugant/donor) ^a^	MICs to QdNOs (µg/ml) ^b^					Similarity of Riboprint patterns before and after transconjugation (%) ^c^
			OLA	MEQ	CAR	CYA	RIF	
Recipient	NK5449		8	4	8	8	>200	100
Donors	79O4-1		256	128	64	>128	12.5	67
	79O4-2		>256	>128	128	>128	12.5	58
	79O4-3		256	128	32	>128	6.25	71
Transconjugant	79O4-1/N	4.0×10^−10^	256	128	128	>128	>200	93
	79O4-2/N	5.4×10^−10^	128	128	128	>128	>200	92
	79O4-3/N	2.5×10^−10^	128	128	128	>128	>200	93

Note: a-the transfer requency was calculated by dividing the CFU of transconjugant by the CFU of donor;

b- and c-the specificity of conjugation was assessed by the change of MIC and riboprint patterns of the recipient, donors and tranconjugants.

### Differentially Expressed Genes Determined by Prokaryotic SSH PCR cDNA Subtraction

By prokaryotic SSH PCR cDNA subtraction, 44 unique fragments were found in the QdNOs resistant strain *E. coli* 79O4-2 comparing to its control strain *E. coli* 79∼20. Among these 44 sequences, 3 sequences were with no significant similarity found in the Genbank; 40 sequences were found in genome of *E. coli*; 1 sequence was found in plasmid of *E. coli* (*mobA*) (see Table 5). The homology search results showed that QdNOs-resistant *E. coli* alters the expression of 18 genes involved in biosynthesis, 11 genes associated with metabolism, 3 genes encoding transporters, 3 genes involved in flagellar assembly, 1 gene encoding mobilization protein and 5 genes associated with hypothetical proteins (see Table 5).

**Table 5:** The differentially expressed fragments identified in the QdNOs resistant strain *E. coli* 79O4-2.

No. of sequence	Length(bp)	Encoded protein	Gene
**Involved in biosynthesis**			
1	435	Iron-sulfur cluster binding protein	-
2	176	Nitrate reductase 1, beta (Fe-S) subunit	*narH*
3	192	RNA polymerase, alpha subunit	*rpoA*
4	88	tRNA (guanine-1-)-methyltransferase	*trmD*
5	112	tRNA pseudouridine synthase A	*truA*
6	147	Glycine tRNA synthetase, beta subunit	*glyS*
7	123	Isoleucyl-tRNA synthetase	*ileS*
8	150	50S ribosomal subunit protein L6	*rplF*
9	126	50S ribosomal subunit protein L3	*rplC*
10	343	50S ribosomal subunit protein L24	*rplX*
11	264	30S ribosomal subunit protein S8	*rpsH*
		50S ribosomal subunit protein L6	*rplF*
12	406	Protein chain elongation factor	*fusA*
13	200	Lipoate synthase	*lipA*
14	233	UDP-3-O-acyl N-acetylglucosamine deacetylase	*lpxC*
15	54	UDP-N-acetyl-D-mannosaminuronic acid transferase	*rffM*
16	170	Enoyl-(acyl-carrier-protein) reductase, ADH-dependent	*fabI*
17	413	Trehalose-6-phosphate synthase	*otsA*
18	66	CTP synthetase	*pyrG*
Involved in metabolism			
19	252	Phosphoglyceromutase 1	*gpmA*
20	473	Glucosephosphate isomerase	*pgi*
21	397	ATP-dependent Clp protease, ATP-binding subunit	*clpX*
22	161	ATP-dependent Clp protease ATP-binding subunit	*clpA*
23	90	Aminopeptidase N	*pepN*
24	137	Peptidyl-prolyl cis-trans isomerase	*fkpB*
25	238	F1 sector of membrane-bound ATP synthase, alpha subunit	*atpA*
26	456	F1 sector of membrane-bound ATP synthase, beta subunit	*atpD*
27	364	F1 sector of membrane-bound ATP synthase, gamma subunit	*atpG*
28	271	2-octaprenylphenol hydroxylase	*ubiB*
29	161	Isochorismatase hydrolase	*yecD*
Involved in transport			
30	170	spermidine/putrescine ABC transporter membrane protein	*potC*
31	441	outer membrane-specific lipoprotein transporter subunit; membranecomponent of ABC superfamily	*lolC*
32	160	Preprotein translocase, SecA subunit	*secA*
Flagellar assembly			
33	232	Flagellar hook assembly protein	*flgD*
34	246	Flagellar biosynthesis protein FlhA	*flhA*
35	544	Flagellin	*fliC*
Mobilization protein			
36	262	Mobilization protein MobA	*mobA*
Predicted protein			
37	111	Putative enzyme	-
38	191	Predicted peptidoglycan peptidase	*yiiX*
39	226	Conserved hypothetical protein	*ytfN*
40	163	Conserved hypothetical protein	*yrbL*
41	325	Conserved hypothetical protein	*ybjP*

Note: – No designated gene name.

**Figure 1:**
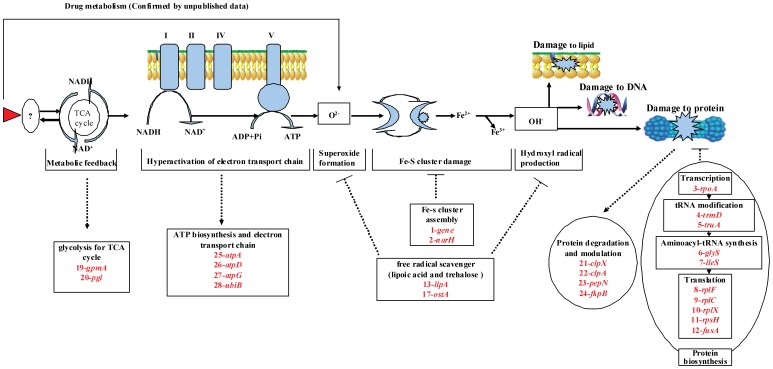
The hyperthetical mechanism of cell death induced by QdNOs and genes involved in the development of Quinoxaline resistance. The primary drug (red triangle)-target (unknown target) interaction initiate a metabolic feedback dependent on tricarboxylic acid (TCA) cycle, stimulate the oxidation of NADH through the electron transport chain, promote superoxide (O^2−^) formation, damage the structure of Fe-S clusters, lead to the formation of hydroxyl radicals (OH^−^); and finally damage DNA, lipids or proteins and contributes to the cell death. The overexpressed genes found in our study (in the box and cycle) were involved into this process. The overexpression of some genes were induced in this drug-target cell death pathway (The dotted arrow), the others may inhibit the formation free radicals or prevent the damage by free radical (dotted line with a bar).

In the 18 genes involved in biosynthesis, the number 1 gene encodes a iron-sulfur cluster binding protein, the number 2 gene (*narH*) encodes a nitrate reductase I. The number 3 to 12 genes (*rpoA, trmD, truA, glyS, ileS, rplFCX, rpsH and fusA*) encode RNA polymerase, tRNA-methyltransferase, tRNA synthase, tRNA synthase and protein chain elongation factor respectively. The number 13 to18 genes (*lipA, lpxC, rffM, fabI, otsA and pyrG*) are related to biosynthesis of lipoate, lipid A, enterobacterial common antigen, fatty acid, trehalose and cytidine triphosphate respectively (see Table 5 and Figure 1).

In the 11 genes involved in metabolism, the number 19 and 20 genes (*gpmA and pgi*) participate in glycogen degradation, the number 21 to 24 genes (*clpX, clpA, pepN and fkpB*) take part in degradation of damaged protein or protein modulation, the number 25 to 29 genes (*atpA, atpD, atpG, ubiB and yecD*) contribute to electron transport and ATP biosynthesis (see Table 5 and Figure 1).

As shown in table 5, the 3 transport proteins include spermidine/putrescine ABC transporter membrane protein (*potC*), outer membrane-specific lipoprotein transporter subunit (*lolC*) and preprotein translocase (*secA*). The *flgD*, *flhA* and *fliC* are involved in assembly of flagellar (see Table 5). The *mobA* gene is associated with the mobilization of bacterial (see Table 5).

**Figure 2:**
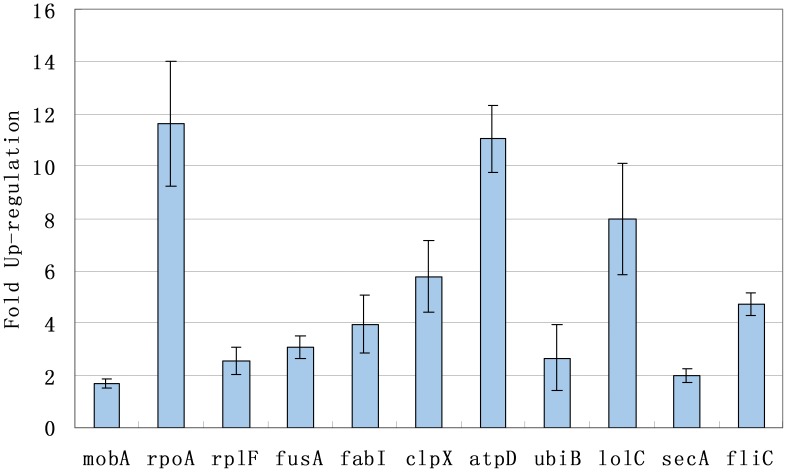
Fold change of 11 up-regulated genes determined by RT-qPCR. The X-axis was the 11 representative genes (*mobA*, *rpoA*, *rplF*, *fusA*, *fabI*, *clpX*, *atpD*, *ubiB*, *lolC*, *secA* and *fliC*) which were selected from all the 44 differentially expressed genes. The Y-axis was the relative fold change of selected genes determined by RT-qPCR. The data were considered for significant differences by one-way ANOVA using SPSS program (P≤0.05).

Up-regulation of 11 respresentative genes (*mobA*, *rpoA*, *rplF*, *fusA*, *fabI*, *clpX*, *atpD*, *ubiB*, *lolC*, *secA* and *fliC*) was confirmed by RT-qPCR. Fold change of the 11 genes is in the range of 1.7 to 11 fold of over-expression with the P value much less than 0.05, indicating that the methodology of prokaryotic SSH PCR cDNA subtraction is reliable to identify differentially expressed genes (see Figure 2).

## Discussion

### Resistance Development Under QdNOs

Although the resistant *E. coli* strains were obtained from step-wise selection by QdNOs drugs *in vitro*, there is no significant change of susceptibility of the *E.coli* strains isolated from pigs farm before and after the usage of QdNOs drugs. It was revealed that the incidence and level of olaquindox resistance in the coliform floras isolated from pigs and pig farms was complex and largely fluctuated, the possible origin of olaquindox-resistant strains may be related to the biotypes of the drug-resistant sub-populations [24]. During the usage of olaquindox as feed additive on commercial pig farms from 1982 to 1984, the monitoring result about the development of olaquindox resistance showed that there was a low but not significant increasing incidence and level of resistance to olaquindox on drug-using farms [25]. Conclusively, it is unreliable to connect the emergency of QdNOs resistance to the usage of QdNOs drugs only by series passage *in vitro*. The resistance of *E.coli* strains isolated from the control group of pigs may due to the complexity of clinical environment.

Compared the MICs of QdNOs in the three strains (79O4-1, 79O4-2 and 79O4-3) selected by olaquindox to the three strains (79C4-1, 79C4-2 and 79C4-3) selected by cyadox, olaquindox appears to possess more potency in selection of cross-resistance to QdNOs, suggesting that long-term use of cyadox could be safer than olaquindox. The difference in the development of cross-resistance induced by olaquindox and cyadox may attribute to the discrepancy in the chemical characteristics or structure of these two drugs [2].

Similar to previous studies of resistant phenotype in *E.coli* strains isolated from pigs and pig farms, the QdNOs resistant strains selected in our study also exhibited multi-drug resistance against tetracycline and chloramphenicol [24], [25], [26]. Both the result of previous studies and our study revealed that multidrug resistance in most of clinical isolates may due to the multidrug efflux pump OqxAB [14], [15]. The OqxAB located plasmid was reported to has a high frequency (10^−4^ CFU/recipient) of transfer among enterobacterial pathogens including *S.Typhimurium and K.pneumoniae, etc* [14], [16]. The E100-4 strain (see table 3) carried the *oqxA* gene but is susceptible to olaquindox, indicating that some strains may acquire the *oqxA* gene from the horizontal transfer in clinical environment. However, different with previous studies, the six QdNOs resistant strains selected by *in vitro* passage ((79O4-1, 79O4-2, 79O4-3, 79C4-1, 79C4-2 and 79C4-3) were negative in OqxAB pump (data not shown ) and showed much lower frequency of horizontal transfer (see table 4) with the ratio of transconjugant to donor only in a range of 2.5∼4.0×10^−10^ CFU/donor, suggesting that other mechanism beyond OqxAB might confer the multidrug resistance and lower transfer risk in the selected strains in our study. A prokaryotic SSH PCR cDNA subtraction was thus carried out to find the novel molecular mechanism involved in QdNOs resistance.

### Differently Expressed Genes Involved in the QdNOs Resistance

Based on networks of the drug-target induced cell death pathway found recently [27], it is supposed that the QdNOs binding to unknown target may initiate a metabolic feedback dependent on the tricarboxylic acid (TCA) cycle, stimulate the oxidation of NADH through the electron transport chain; promote superoxide (O^2−^) formation; damage the structure of Fe-S clusters, lead to the formation of hydroxyl radicals (OH^−^); and finally damage DNA, lipids or protein and contributes to the death of *E.coli* (see Figure 1).

In the differently expressed genes, the iron-sulfur cluster binding protein encoding gene (gene number 1) participates in Fe-S cluster assembly. The nitrate reductase 1 encoding gene (*narH*) contribute to the electron transfer for Fe-S cluster [28], [29]. Thus, overexpression of number 1 and 2 genes (*narH*) may compensate Fe-S cluster damage by assembly of Fe-S cluster (see figure 1).

The *rpoA* is a DNA-direct RNA polymerase which can interact with other proteins (*rplF*, *rplC*, *rplX*, *rpsH* and others) and plays importance role in transcriptional regulation [30], [31]. The *trmD, truA*, *glyS and ileS* are essential enzymes for tRNA modification and aminoacyl-tRNA synthesis which also play essential role on the protein synthesis [32]. The *fusA* gene encoding for a translational regulator which is essential for translational elongation [33], [34]. The overexpression of the translational regulator *fusA* and some ribosomal proteins involved in translational process may compensate protein synthesis for oxidative damage by QdNOs and contribute to the multidrug resistance against some protein target drug of tetracycline and chloramphenicol.

The number 13 gene (*lipA*) encodes lipoate synthase (LS) which is involved in the biosynthesis of Lipoic acid [35], [36], [37]. Lipoic acid is a protein-bound dithiol- containing cofactor which is commonly required for the energy metabolism in *E. coli* [37]. Recent studies also show that lipoic acid is a free radical scavenger and a potent antioxidant [38], [39], [40]. The number 17 gene (*otsAB*) encodes trehalose-6-phosphate synthase which is responsible for trehalose biosynthesis [41], [42], [43]. It has been shown that trehalose can protect proteins and cellular membranes from inactivation or denaturation caused by a variety of stress conditions, including desiccation, dehydration, heat, cold or oxidation [42], [43], [44]. The accumulation of trehalose can protect *Saccharomyces cerevisiae* from oxidative damage and enhance resistance of bacterial to H_2_O_2_, indicating that trehalose may be a good free radical scavenger [45]. Therefore, the overexpression of *lipA* and *otsA* gene may promote production of two free radical scavenger (lipoic acid and trehalose) which may protect QdNOs-resistant *E. coli* from oxidative damage and result in QdNOs-resistance. There is no significant change of the expression of *oxyR* or *soxRS* regulon, so this mechanism of antioxidative protection mediated by lipoic acid and trehalose may be independent on the regulation of *oxyR* or *soxRS* [46].

The number 19 gene (phosphoglyceromutase 1, *gpmA*) and number 20 gene (glucosephosphate isomerase, *pgi*) are involved in glycolysis which generates a small quantity of ATP and degrades glucose into pyroracemic acid and acetyl-CoA for tricarboxylic acid cycle (TCA) under aerobic growth condition. These two enzymes encoded by *gpmA* and *pgi* gene are also important for central carbon metabolism (CCM) pathway which may influence the fidelity of DNA replication in *E.coli* [47], [48]. The 2-octaprenylphenol hydroxylase (*ubiB*), F1 sector of membrane-bound ATP synthase α (*atpA*), β *(atpD*) and γ (*atpG*) subunit are involved in ATP biosynthesis, electron transport chain and oxidative phosphorylation [49].

Based on the function annotation and pathway analysis, the Clp, one of the key ATP-dependent proteases in *E. coli*, is required during some stress conditions [50], [51], [52], [53]. The PepN, a major aminopeptidase in *E. coli*, can degrade amino acids into small molecular for TCA cycle [54]. The Clp protease cleave damaged proteins into large peptides and amino acids which are further degraded by PepN [55]. The *fkpB*, peptidyl-prolyl cis-trans isomerase, participates in several biochemical processes including protein folding, receptors signaling, protein trafficking and transcription [56]. Therefore, up-regulation of Clp proteases (21-ClpX and 22-ClpA), aminopeptidase pepN (number 23 gene)and protein folding chaperones *fkpB* (number 24 gene)may play important roles in regulating levels of specific proteins and in eliminating or modifying damaged or abnormal proteins [57].

Considering the drug-target induced cell death pathway and function analysis of the differentially expressed genes, the upregulation of some genes may be response for some process of cell death pathway, but others may compensate or inhibit that pathway. See figure 1, the two genes involved in glycolysis for TCA cycle (1*9-gpmA and 20-pgi*), four genes related to ATP biosynthesis and electron transport chain (25-atpA, 26-atpD, 27-atpG and 28-ubiB) and four genes associated with protein degradation and modulation (*21-clpX, 22-clpA, 23-pepN and 24-fkpB*) might be response for the metabolic feedback, hyperactivation of electron transport chain and protein damage, respectively. However, the two genes involved in Fe-S cluster assembly (*1-gene and 2-narH*) and several genes associated with protein biosynthesis may compensate the Fe-S cluster damage and protein damage respectively (see Figure 1). The overexpression of genes involved in biosynthesis of lipoic acid and trehalose (*13-lipA and ostA*) may eliminate superoxide formation and hydroxyl radical production (see Figure 1). The role of *lipA* and *ostA* on clearage of free radical and development of QdNOs resistance would be investigated in our next plan, because unpublished data obtained in our lab recently revealed that QdNOs are endowed with producing free radical in *E.coli* cell.

Additionally, the *potC* gene is involved in spermidine uptake which plays an important role in bacterial growth including cell proliferation and differentiation [58]. The *lolC* gene encodes an outer membrane-specific lipoprotein transporter which mediates the detachment of lipid-modified proteins and catalyzes release of outer membrane-directed lipoproteins from the inner membrane of *E. coli* [59], [60], [61]. Disruption of *lolCDE* can prevent release of lipoprotein from inner membrane and shows to be lethal for *E.coli* [*60*] *.* The *secA* gene encodes a peripheral membrane ATPase which participates in preproteins translocation across the bacterial cytoplamic membrane and plays an essential role in the survival of the bacterial species [62]. Recent study demonstrated that export of the superoxide dismutase SodA to periplasm was dependent on activity of SecA ATPase [63]. Therefore, overexpression of genes involved in transporters plays an essential role on the survival and growth of QdNOs-resistant *E. coli*. The *lpxC* encodes zinc-dependent UDP-3-O-(acyl)-N-acetylglucosamine deacetylase which catalyzes the first irreversible step of lipid A biosynthesis [64]. Lipid A biosynthesis (*lpxC*), cytidine triphosphate biosynthesis (*pyrG*), fatty acid biosynthesis (*fabI*) and enterobacterial common antigen biosynthesis (*rffM*) may be essential for viability of *E.coli*. The *flgD*, *flhA and fliC* genes are involved in flagellar assembly which exhibit functions in cell motility and sensibility [65]. Recent findings revealed that the flagellum participated in a variety of processes including biofilm formation, pathogenesis and even regulation of gene expression [58]. The *mobA* gene encodes a mobilization protein which can be induced by some drugs and mediates the transfer of bacteroides conjugation [66], [67]. These genes may play important roles in physiological functions of QdNOs-resistant *E. coli*.

### Conclusions

Results of the present study suggest that olaquindox possess more potency in selection of multi-drug resistant and cross-resistant *E. coli* strains. The induced QdNOs-resistant determinant shows lower frequencies of conjugation than previous reports in clinical isolates. More than 90% clinical isolates carry *oqxA* gene, but it is negative in our *in vitro* selected QdNOs-resistant strains, indicating that there might be other mechanism conferring resistance. By SSH PCR subtraction, 44 genes were differentially expressed in the QdNOs-resistant strain 79O4-2 selected by olaquindox in vitro. The overexpression of genes involved in glycolysis for TCA cycle (*gpmA and pgi*) and genes involved in ATP biosynthesis and electron transport chain (*atpADG and ubiB*) may due to the metabolic feedback and hyperactivation of electron transport chain, respectively. The genes involved in Fe-S cluster assembly (1-gene and *narH*) and genes involved in biosynthesis of lipoic and trehalose (*lipA* and *ostA*) may play an important role in clearage of free radical and prevention of Fe-S damage. The genes involved in protein biosynthesis (*rpoA*, *trmD*, *truA*, *glyS*, *ileS*, *rplF*, *rplC*, *rplX*, *rpsH*, *rplF* and *fusA*) and genes involved in protein degration and modulation may participate in compensation of the protein damage (see Figure 1). The other genes including genes encoding transporter, flagellar, lipid A and mobilization protein may be essential for the growth and physiological functions of QdNOs-resistant *E. coli.* Conclusively, most of these genes have previously been implicated in resistance or tolerance to superoxide stress, suggesting that their altered expression may be a part of a protective response in QdNOs-resistant *E. coli* [68]. Likewise, other identified changes in gene expression may affect the growth and transfer capacity of QdNOs resistance. Further studies are required to investigate the roles of these genes in development of QdNOs resistance by technique of gene knockout and transcriptomics or proteomics. In addition, the molecular mechanism involved in the discrepancy of resistance development selected by olaqindox and cyadox is necessary to be investigated in future, because these information will be useful for the designation of new member of versatile QdNOs.
